# Association of *VEGFA*-2578 C>A polymorphism with clinicopathological aspects and outcome in follicular lymphoma patients

**DOI:** 10.1038/bcj.2016.76

**Published:** 2016-08-26

**Authors:** G R A de Mendonça, A B C Brito, R M Rocha, M T Delamain, R de Andrade Natal, F A Soares, G W B Colleoni, C A Souza, J Vassallo, C S P Lima

**Affiliations:** 1 Faculty of Medical Sciences, University of Campinas, Campinas, Brazil; 2 Department of Pathology, AC. Camargo Cancer Center, São Paulo, Brazil; 3 Hematology and Hemotherapy Center, University of Campinas, Campinas, Brazil; 4 Department of Clinical and Experimental Oncology, Federal University of São Paulo, São Paulo, Brazil; 5 Laboratory of Molecular and Investigative Pathology, Faculty of Medical Sciences, University of Campinas, Campinas, Brazil

Angiogenesis (AG) based on immunohistochemistry (IHC) for related proteins is a rather contradictory prognostic marker in follicular lymphoma (FL).^1, 2^ These inconsistencies may result from the variety of treatments in different cohorts, and also from the complex tumor–host interaction in this disease.^3^

The vascular endothelial growth factor A (VEGFA), produced both by lymphoma and microenvironment cells, mediates AG^4^ and seems to interfere in lymphangiogenesis (LG) in lymphomas.^5^ A single-nucleotide polymorphism (SNP) in the promoter region of the *VEGFA* gene, with a C→A substitution at −2578 nucleotide position (rs699947), had its wild-type genotype CC associated with a higher protein expression when compared with the CA or AA genotypes in immunoassay-based studies.^6, 7, 8^ More recently, the *VEGFA* CC genotype was associated with increased risks of mantle cell lymphoma (MCL)^9^ and multiple myeloma,^10^ suggesting an influence of this locus in the development of mature lymphoplasmacytic neoplasias. The SNP did not influence the survival of MCL^11^ and diffuse large B-cell lymphoma (DLBCL) patients,^12^ but a *VEGFA* haplotype, including the rs699947 locus, determined worse prognosis in chronic lymphocytic leukemia (CLL) patients.^13^ To the best of our knowledge, the roles of *VEGFA*-2578 C>A SNP in FL susceptibility, clinicopathological features and outcome are still unknown, and, therefore, these were the aims of this study. Moreover, we investigated a possible functional role of rs699947 in FL, by combining genotyping with the analysis of AG and LG markers in the patients' biopsies.

Newly diagnosed FL patients (*N*=171; median age: 56 years; 78 males, 93 females; 99 of histological grade 1 or 2, 30 of grade 3A, 42 unclassified; 41 of Ann Arbor stage I or II, 130 of III or IV) and controls (209 blood donors; median age: 50 years; 103 males, 106 females) were included in the study from January 1999 to December 2014 (Supplementary Table S1), after Ethics Committees approvals. Patients received CHOP (*N*=24), R-CHOP (*N*=115) or other regimens (*N*=17) as induction therapy; 15 patients were maintained in watch and wait regimen.

Genotypes were identified in DNA of peripheral blood by real-time polymerase chain reaction, using a Taqman SNP Genotyping Assay (catalog #4351379, ThermoFisher Scientific, Foster City, CA, USA). Replicates were performed in 10% of the reactions, achieving 100% of concordance. Eighty-two patients' diagnostic paraffin blocks, arranged as a tissue microarray, served for IHC analyses using anti-VEGFA, anti-CD34 and anti-D2-40. The slides were scanned at 20 × magnification in Aperio Scanscope XT (Aperio Technologies, Vista, CA, USA) and submitted to algorithms for analysis (Supplementary Table S2), in a blinded fashion.

The Hardy-Weinberg equilibrium (HWE) was verified using *χ*^*2*^ statistics for the goodness-of-fit. Differences between groups were analyzed by the *χ^2^* or Fisher's exact test. Two-tailed *t*-tests were performed to compare IHC scores between groups of patients. Logistic regression models assessed associations between genotypes and clinicopathological features. Event-free survival (EFS) and overall survival (OS) encompassed time from diagnosis until relapse, progressive disease, death due to disease effects or last follow-up, and time from diagnosis until death by any cause or last follow-up, respectively, and were considered only in the 139 patients treated with CHOP or R-CHOP. EFS and OS probabilities were estimated by the Kaplan–Meier method, and curves were compared by the log-rank test. The Cox hazards model was used to identify variables predicting EFS and OS. Variables with *P*<0.15 in univariate analyses were included in multivariate analysis. Significant results of Cox analyses were validated using a bootstrap resampling study to investigate the stability of risk estimates (1000 replications). Differences were significant when *P*<0.05.

Both patients (*χ*^*2*^=0.0002, *P=*0.98) and controls (*χ*^2^=1.73, *P*=0.18) satisfied the HWE at the *VEGFA*-2578 C>A locus. The mean age of patients was higher than that of controls (56 vs 50 years, *P*<0.001). Similar frequencies of *VEGFA* CC genotype were seen in patients and controls (39.2% vs 36.8%, *P*=0.34), after adjustment by differences in age of groups, and individuals with the CC genotype were under similar risks of FL than the ones carrying CA or AA genotypes (odds ratio: 1.12, 95% confidence interval (CI): 0.72–1.75). However, patients harboring the *VEGFA* CC genotype had 2.11- (95% CI: 1.11–4.02) and 2.34 (95% CI: 1.10–4.97)-fold increased chances of presenting B symptoms and belonging to high or intermediate Follicular Lymphoma International Prognostic Index (FLIPI) groups, respectively (Supplementary Table S1).

VEGFA and D240 protein expressions in tumor samples were similar in patients with distinct SNP genotypes (data not shown), but FL microvessel density was increased in patients with the CC genotype compared with others (3.3 × 10^−4^ vs 2.4 × 10^−4^ vessels/μm^2^) (Figures 1a–c).

At the study end (May 2016), 116 patients were alive and 23 patients had died. The median follow-up time was 41.0 months (1.7–196.1). The 60-month EFS and OS for all patients were 56.5% and 80.5%, respectively. At this time, both EFS and OS were shorter in patients with B symptoms (40.9% vs 67.9%, *P*<0.01; 66.4% vs 90.1%, *P*<0.01), FLIPI of high risk (33.2% vs 68.3%, *P*<0.01; 59.4% vs 91.0%, *P*<0.01) and *VEGFA* CC genotype (42.4% vs 68.3%, *P*=0.02; 65.3% vs 91.1%, *P*<0.01), respectively (Figures 1d and e). Bulky disease predicted only worse OS (65.0% vs 87.7%, *P*=0.03), and bone marrow infiltration was marginally associated with shorter EFS (37.0% vs 59.6%, *P*=0.05). No differences in EFS or OS were seen in patients treated with CHOP or R-CHOP (Figures 1f and g) (Kaplan–Meier estimates). In univariate Cox analysis, B symptoms, FLIPI score and *VEGFA*-2578 C>A predicted EFS and OS, and bulky disease influenced only OS. In multivariate analysis, B symptoms predicted EFS, bulky disease predicted OS and *VEGFA*-2578 C>A influenced both EFS and OS. Univariate and multivariate analyses showed that patients with the *VEGFA* CC genotype had a 1.84- and 1.82-fold higher risk of presenting an event and a 3.76- and 3.35-fold increased risk of progression to death than others, respectively. All associations seen in Cox analyses were validated by bootstrap studies based on 1000 samples (Table 1).

We initially observed that the rs699947 SNP was unimportant in FL development, but had a relevant role in determining tumor burden, aggressiveness and vascularization at diagnosis. In fact, increased blood vessels may determine non-Hodgkin lymphoma progression, probably due to increased supplies of oxygen and nutrients to tumor cells.^2, 4^

Second, we found lower EFS and OS in patients with B symptoms, bulky disease and FLIPI of high risk, as expected, and observed that patients with the *VEGFA* CC genotype presented poorer outcome than others. No association between rs699947 and patients' outcome was found in a large study in DLBCL and in a small MCL cohort.^11, 12^ In contrast, a *VEGFA* haplotype, including the rs699947 locus, had deleterious effects in CLL patients.^13^ Our results and those found in CLL support that AG modulation by rs699947 may be relevant driving indolent lymphoproliferative diseases to unfavorable outcomes.

The *VEGFA* CC genotype was previously associated with higher protein expression in the peripheral blood of 30 healthy individuals^7^ and 52 ovarian cancer patients.^8^ We found, however, similar VEGFA expressions in tumors derived from FL patients with distinct genotypes, suggesting that the SNP does not affect protein levels. Interestingly, the final AG product, that is, microvessel density, was increased in biopsies of patients carrying the CC genotype. It seems, thus, that the functional role of rs699947 also exists in FL, but is not dependent on local VEGFA production, as similarly reported in other AG studies for FL.^5, 14^ It is possible, however, that rs699947 affects systemic VEGFA levels in FL patients, as described in the abovementioned immunoassay studies.^7, 8^ Complementarily, the lack of LG modulation by *VEGFA*-2578 C>A in our study suggests an exclusive role of the CC genotype in AG, which could increase resistance to apoptosis and tumor cell migration, leading ultimately to disease progression, as previously modeled.^13^ Another report, in contrast, observed that higher VEGFA levels in lymphoma samples were correlated with increased LG.^5^ However, only 11 FL cases were analyzed by the authors, hampering a more direct comparison with our results. In addition, our findings do not support a tumor VEGFA modulation dependent on rs699947. Growing evidences, however, point toward regulation of LG by the *VEGFC* gene, which might guide future investigations.^15^

In summary, we present the first evidence regarding *VEGFA*-2578 C>A SNP role in clinicopathological aspects and as an independent prognostic factor in FL. However, we are aware that larger cohorts of various parts of the world and studies of systemic VEGFA production in patients carrying the different genotypes of the SNP are needed to give support to the findings.

## Figures and Tables

**Figure 1:**
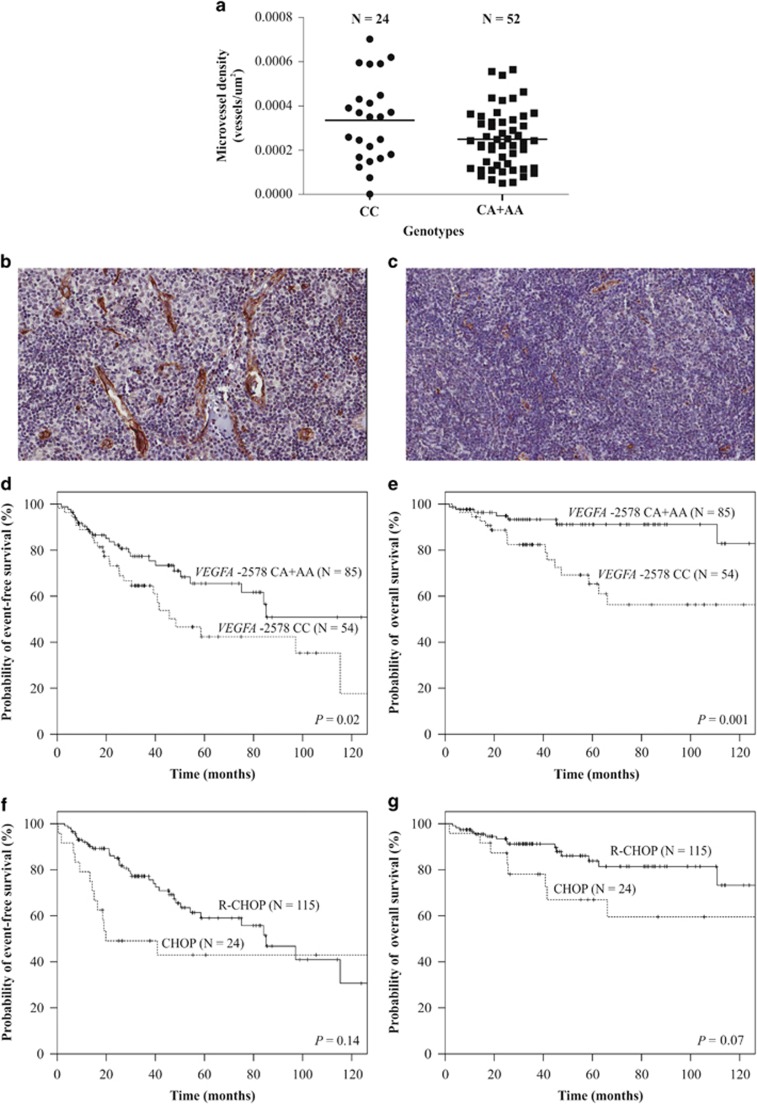
(**a**) Represents microvessel density measured by CD34 staining in biopsies from CC vs CA+AA patients (*P*=0.02, two-tailed *t*-test); six cases out of 82 showed technical unsuitability for microvessel analysis. (**b**, **c**) Show extreme images of cases with the respective abovementioned genotypes with high and low microvascular densities, respectively (CD34 staining, 20 × magnification). (**d**, **e**) Represent the estimated EFS and OS of FL patients treated with CHOP/R-CHOP stratified by the VEGF-2578 C>A genotypes. (**f**, **g**) Show the estimated EFS and OS of FL patients submitted to CHOP or R-CHOP regimens in first-line treatment.

**Table 1:** Association of clinicopathological features and *VEGFA*-2578 C>A genotypes with survival in follicular lymphoma patients

*Characteristics*	*Univariate Cox analysis*					*Multivariate Cox analysis*			
	*N (%)*	*EFS HR (95% CI)*	P-*value*	*OS HR (95% CI)*	P- *value*	*EFS HR (95% CI)*	P-*value*	*OS HR (95% CI)*	P*-value*
*B symptoms*									
Present	55	2.76 (1.58–4.80)	**<0.01**^a^	3.85 (1.58–9.38)	**<0.01**^b^	2.18 (1.15–4.11)	**0.01**^c^	2.96 (0.98–8.90)	0.05
Absent	84	Reference		Reference		Reference		Reference	
									
*Bulky disease*^d^									
Present	39	0.96 (0.51–1.82)	0.92	2.38 (1.02–5.53)	**0.04**^e^	NA	NA	2.59 (1.01–6.63)	**0.04**^f^
Absent	94	Reference		Reference				Reference	
									
*Bone marrow infiltration*									
Present	64	1.70 (0.98–2.97)	0.05	1.44 (0.62–3.31)	0.38	1.38 (0.75–2.54)	0.28	Not considered	NA
Absent	75	Reference		Reference		Reference		Reference	
									
*FLIPI score*^d^									
High risk	41	2.24 (1.26–3.95)	**<0.01**^g^	5.01 (2.01–12.44)	**<0.01**^h^	1.55 (0.83–2.89)	0.16	2.55 (0.93–6.96)	0.06
Low/intermediate risk	96	Reference		Reference		Reference		Reference	
									
*Treatment*									
CHOP	24	1.61 (0.84–3.08)	0.14	2.21 (0.93–5.24)	0.07	1.12 (0.55–2.25)	0.74	1.52 (0.58–4.00)	0.39
R-CHOP	115	Reference		Reference		Reference		Reference	
									
*VEGFA-2578 C>A*									
CC	54	1.84 (1.06–3.17)	**0.02**^i^	3.76 (1.54–9.16)	**<0.01**^j^	1.82 (1.02–3.24)	**0.04**^k^	3.35 (1.22–9.12)	**0.01**^l^
CA+AA	85	Reference		Reference		Reference		Reference	

Abbreviations: CI, confidence interval; EFS, event-free survival; FLIPI, Follicular Lymphoma International Prognostic Index; HR, hazard ratio; N, number of patients; NA, not applicable; OS, overall survival; %, percentage.

In univariate and multivariate Cox analysis:

a Pbootstrap<0.01,

b Pbootstrap<0.01,

c Pbootstrap=0.01,

d The number of patients differed from the total treated with CHOP/R-CHOP included in analysis of survival (*N*=139) because it was not possible to obtain the information of interest in some patients.

e Pbootstrap=0.03,

f Pbootstrap=0.04,

g Pbootstrap<0.01,

h Pbootstrap<0.01,

i Pbootstrap=0.02,

j Pbootstrap<0.01,

k Pbootstrap=0.04,

l Pbootstrap=0.02.

Significant differences between groups are presented in bold letters.
