# Supplementary material for: Association of VEGFA-2578 C>A polymorphism with clinicopathological aspects and outcome in follicular lymphoma patients
Source: Blood Cancer J. 2016 Aug 26;6(8):e464–. doi: 10.1038/bcj.2016.76 (PMC5022185; doi:10.1038/bcj.2016.76)
Supplement: Supplementary Tables [file bcj201676x1.doc]

**Table S1.** Clinicopathological features and genotypes in follicular lymphoma patients and controls.

| **Variable** | **N (%)** |  | ***VEGFA* -2578 C>A genotypes** | | | | |
| --- | --- | --- | --- | --- | --- | --- | --- |
|  |  |  | **CC** | **CA** | **AA** | ***P* value** | |
| **N (patients)** | 171 |  | 65 (38.0) | 82 (48.0) | 24 (14.0) |  | |
| **Median age (range) in years** | 56 (18–94) |  | 56 (18–93) | 55 (21–87) | 54.5 (25–94) | 0.40 | |
| **Mean age (SD)** | 55.6 (12.9) |  | 55.5 (13.8) | 55.8 (11.3) | 55.0 (15.9) | 0.46 | |
| **Gender** |  |  |  |  |  |  | |
| Male | 78 (45.6) |  | 30 ( 38.5) | 37 (47.4) | 11 (14.1) | 0.90 | |
| Female | 93 (54.4) |  | 35 (37.6) | 45 (48.4) | 13 (14.0) |  |  |
| **B symptoms** |  |  |  |  |  | |  |
| Absent | 108 (63.1) |  | 34 (31.5) | 57 (52.8) | 17 (15.7) | **0.02** | |
| Present | 63 (36.9) |  | 31 (49.2) | 25 (39.7) | 7 (11.1) |  |  |
| **Bulky disease*** |  |  |  |  |  |  | |
| No | 112 (72.7) |  | 44 (39.3) | 54 (48.2) | 14 (12.5) | 0.34 | |
| Yes | 42 (27.3) |  | 13 (31.0) | 21 (50.0) | 8 (19.0) |  |  |
| **Bone marrow infiltration** |  |  |  |  |  |  | |
| No | 93 (54.4) |  | 36 (38.7) | 47 (50.5) | 10 (10.8) | 0.82 | |
| Yes | 78 (45.6) |  | 29 (37.2) | 35 (44.9) | 14 (17.9) |  |  |
| **Histological grade*** |  |  |  |  |  |  | |
| 1 or 2 | 99 (76.7) |  | 26 (26.3) | 57 (57.6) | 16 (16.1) | 0.73 | |
| 3A | 30 (23.3) |  | 6 (20.0) | 17 (56.7) | 7 (23.3) |  |  |
| **Stage (Ann Arbor)** |  |  |  |  |  |  | |
| I/II | 41 (24.0) |  | 13 (31.7) | 24 (58.5) | 4 (9.8) | 0.30 | |
| III/IV | 130 (76.0) |  | 52 (40.0) | 58 (44.6) | 20 (15.4) |  |  |
| **FLIPI score*** |  |  |  |  |  |  | |
| Low risk | 49 (29.5) |  | 12 (24.5) | 30 (61.2) | 7 (14.3) | **0.02** | |
| Intermediate/high risk | 117 (70.5) |  | 50 (42.7) | 50 (42.7) | 17 (14.6) |  |  |
| **Treatment*** |  |  |  |  |  |  | |
| CHOP | 24 (14.0) |  | 12 (50.0) | 8 (33.3) | 4 (16.7) | 0.22 | |
| R-CHOP | 115 (67.25) |  | 41 (35.7) | 59 (51.3) | 15 (13.0) |  | |
|  |  |  |  |  |  |  | |
| **N (controls)** | 209 |  | 77 (36.9) | 92 (44.0) | 40 (19.1) |  | |
| **Median age (range)** | 50 (18–64) |  | 50 (18–60) | 51 (21–64) | 48 (22–58) | 0.63 | |
| **Mean age (SD)** | 48.6 (8.3) |  | 48.0 (8.5) | 49.4 (8.6) | 47.7 (7.1) | 0.81 | |
| **Gender** |  |  |  |  |  |  | |
| Male (%) | 103 (49.3) |  | 42 (40.8) | 45 (43.7) | 16 (15.5) | 0.63 | |
| Female (%) | 106 (50.7) |  | 35 (33.0) | 47 (44.3) | 24 (22.7) |  | |

N, number of cases; %, percentage; SD, standard deviation; FLIPI, Follicular Lymphoma International Prognostic Index; CHOP/R-CHOP, cyclophosphamide, doxorubicin, vincristine, prednisolone with and without rituximab.

The values in parenthesis are range, SD or percentage. All comparisons were performed between individuals carrying the CC genotype *versus* the ones carrying CA or AA genotypes.

*The numbers of patients differed from the total quoted in the study (N= 171) because it was not possible to obtain consistent information about bulky disease, histological grade and FLIPI score in some cases. *P* values < 0.05 are presented in bold letters.

**Table S2.** Antibodies and quantification methods for immunohistochemical reactions.

| **Antibody** | **Clone** | **Stained elements** | **Algorithm for analysis** | **Algorithm input** | **Methodology of quantification** |
| --- | --- | --- | --- | --- | --- |
| Anti-VEGFA | SP28, ThermoFisher Scientific | Lymphoma and microenvironment cells | Aperio® Positive Pixel Count | Hue value = 0.1  Hue width = 0.5  CST = 0.1 | H-score* |
| Anti-D240 | D2-40,  Dako | Lymphatic vessels | Aperio® Positive Pixel Count | Hue value = 0.1  Hue width = 0.5  CST = 0.1 | Fraction of positive-stained pixels** |
| Anti-CD34 | QBEnd/10,  BioSB | Blood vessels | Aperio® Microvessel Analysis | Mode = 1  DST = 165  LST = 200  RJP (µm) = 20  VCP (µm) = 20 | Microvessel density |

CST, color saturation threshold; DST, dark staining threshold; LST, light staining threshold; RJP, region joining parameter; VCP, vessel completion parameter.

*H-score was calculated as applied by Byers et al. (Blood 2011; 118:2857–2867).

**Any intensity of positivity was considered for calculation.
